# Magnetic Resonance Imaging-Guided High-Intensity Focused Ultrasound Ablation of Uterine Fibroids: Effect of Bowel Interposition on Procedure Feasibility and a Unique Bowel Displacement Technique

**DOI:** 10.1371/journal.pone.0155670

**Published:** 2016-05-17

**Authors:** Young-sun Kim, Hyo Keun Lim, Hyunchul Rhim

**Affiliations:** 1 Department of Radiology and Center for Imaging Science, Samsung Medical Center, Sungkyunkwan University School of Medicine, Seoul, Korea; 2 Department of Health Science and Technology, Samsung Advanced Institute for Health Sciences and Technology, Sungkyunkwan University School of Medicine, Seoul, Korea; University of Chicago, UNITED STATES

## Abstract

**Purpose:**

To evaluate the effect of bowel interposition on assessing procedure feasibility, and the usefulness and limiting conditions of bowel displacement techniques in magnetic resonance imaging-guided high-intensity focused ultrasound (MR-HIFU) ablation of uterine fibroids.

**Materials and Methods:**

Institutional review board approved this study. A total of 375 screening MR exams and 206 MR-HIFU ablations for symptomatic uterine fibroids performed between August 2010 and March 2015 were retrospectively analyzed. The effect of bowel interposition on procedure feasibility was assessed by comparing pass rates in periods before and after adopting a unique bowel displacement technique (bladder filling, rectal filling and subsequent bladder emptying; BRB maneuver). Risk factors for BRB failure were evaluated using logistic regression analysis.

**Results:**

Overall pass rates of pre- and post-BRB periods were 59.0% (98/166) and 71.7% (150/209), and in bowel-interposed cases they were 14.6% (7/48) and 76.4% (55/72), respectively. BRB maneuver was technically successful in 81.7% (49/60). Through-the-bladder sonication was effective in eight of eleven BRB failure cases, thus MR-HIFU could be initiated in 95.0% (57/60). A small uterus on treatment day was the only significant risk factor for BRB failure (B = 0.111, *P* = 0.017).

**Conclusion:**

The BRB maneuver greatly reduces the fraction of patients deemed ineligible for MR-HIFU ablation of uterine fibroids due to interposed bowels, although care is needed when the uterus is small.

## Introduction

Magnetic resonance imaging-guided high-intensity focused ultrasound (MR-HIFU) ablation has been increasingly adopted worldwide as a non-surgical therapy for symptomatic uterine fibroids, due to its satisfactory therapeutic efficacy in controlling symptoms and its high level of safety [1–4]. MR-HIFU ablation can be performed in a totally non-invasive manner, preventing scarring and bleeding, and even hospitalization.

Nonetheless, MR-HIFU ablation therapy cannot be used for all patients due to a number of limiting factors, and 14–74% of referred patients were reportedly eligible for this procedure [5–7]. One of these limiting factors is bowel interposition between the abdominal wall and the uterus, blocking the sonication path. Bowel interposition during HIFU ablation carries a potential risk of bowel perforation and peritonitis due to near-field heating, which might be potentiated by bowel gas, and could damage the bowel wall [8]. Therefore, for a safe procedure, it is extremely important to take the interposed bowel loops out of the beam path before initiating HIFU sonication.

Because manual or instrumental manipulation of the uterus is not possible in the bore of an MR scanner, a number of methods have been suggested for displacing the interposed bowel loops, such as urinary bladder filling with saline, rectal filling with ultrasound gel, and the use of a convex gel pad [9]. Of these, sequential applications of urinary bladder filling, rectal filling, and urinary bladder emptying (*i*.*e*., the BRB maneuver) were reported effective [10]. However, according to accumulation of the experiences, it has become clear that there are additional, important aspects of this bowel displacement technique that need to be further reported, including the effectiveness in retroverted/flexed uterus, technical tips and limiting conditions.

The purpose of our study was therefore to evaluate the influence of bowel interposition on assessing procedure feasibility with or without the BRB maneuver, and to determine the usefulness and limiting conditions of this bowel displacement technique in the MR-HIFU ablation of uterine fibroids.

## Materials and Methods

### Patients

This study was approved by the institutional review board of Samsung Medical Center, Seoul, Korea, and the need for patient consent for this study was waived by the institutional review board because this was a retrospective clinical study. Informed consent for MR-HIFU ablation procedure was obtained from all patients.

Between September 2010 and December 2014, 375 women (mean age, 43.2 years; range, 25–55 years) underwent screening MR exams for MR-HIFU ablation of uterine fibroids. Women were considered eligible for the procedure if they were: 1) premenopausal or perimenopausal and aged 18–59 years; 2) not pregnant; 3) not contraindicated for MRI or the contrast agent; 4) without MRI findings that would prevent the procedure [11]; and 5) without evidence of calcification or degeneration (over 50% in volume) of the fibroid(s). The patient’s decision to undergo MR-HIFU ablation instead of surgery or uterine artery embolization was guided by their physician. MR-HIFU ablation could not be performed in 127 of 375 cases (33.9%), for reasons (multiple reasons in some cases) including a deep fibroid location (n = 45), bowel interposition (n = 43), very large and/or numerous fibroid(s) (n = 36), excessive T2 high signal intensity and/or perfusion (n = 27), an unavoidable susceptible scar (n = 10), substantial fibroid degeneration (n = 10), a very thick abdominal wall subcutaneous fat layer (n = 4), a suspicion of other pathologies (n = 2), surgical material in the sonication path (n = 1), and too high location of the fibroid (n = 1).

Between November 2010 and March 2015, 206 of 248 eligible women underwent MR-HIFU ablation (mean age, 43.0 years; range 22–55 years). The remaining 41 patients delayed the treatment or chose other modalities on their own will. Baseline patient characteristics are summarized in Table 1.

**Table 1:** Baseline Features of the Study Population.

Characteristics	MR-HIFU Ablation	MR-HIFU Ablation with BRB Maneuver
***Patients***	*n = 206*	*n = 60*
Age (years)	43.2 ± 4.8 (25–55)	42.2 ± 5.1 (28–53)
Body mass index (kg/m^2^)	22.2 ± 2.7 (16.2–30.8)	21.7 ± 2.5 (16.2–28.1)
History of pregnancy	66.0% (136/206)	60.0% (36/60)
History of full-term delivery	62.1% (128/206)	63.3% (38/60)
History of Cesarean section	17.5% (36/206)	13.3% (8/60)
Uterine configuration		
Forward-bent	93.2% (192/206)	81.7% (49/60)
Backward-bent	6.8% (14/206)	18.3% (11/60)
Uterine size on screening day (cm)	12.2 ± 2.7 (7.8–19.5)	10.3 ± 1.4 (7.8–13.9)
Uterine size on treatment day (cm)	11.9 ± 2.7 (6.8–19.0)	9.9 ± 1.6 (6.8–14.0)
Pretreatment with GnRH agonist	16.0% (33/206))	28.3% (17/60)
History of fibroid treatment	21.8% (45/206)	26.7% (16/60)
Medical therapy*	11.2% (23/206)	15.0% (9/60)
Myomectomy	6.3% (13/206)	10.0% (6/60)
Radiofrequency ablation	1.5% (3/206)	1.7% (1/60)
Uterine fibroid embolization	1.5% (3/206)	0% (0/60)
Ultrasound-guided HIFU	1.5% (3/206)	0% (0/60)
Symptom severity score^†^	46.1 ± 18.8 (9.4–125)	49.7 ± 20.8 (9.4–93.8)
Number of fibroids treated	1.9 ± 1.4 (1–8)	1.8 ± 1.4 (1–6)
***Fibroids***	*n = 401*	*n = 106*
Diameter (cm)	6.1 ± 3.4 (2.0–16.0)	5.7 ± 2.1 (2.0–11.6)
Type		
Location		
Intramural	38.7% (155/401)	35.8% (38/106)
Subserosal	24.9% (100/401)	26.4% (28/106)
Submucosal	27.7% (111/401)	27.4% (29/106)
Transmural	8.7% (35/401)	10.4% (11/106)
Signal on T2-weighted MR image^‡^		
Type I	59.9% (240/401)	61.3% (65/106)
Type II	32.2% (129/401)	31.1% (33/106)
Type III	8.0% (32/401)	7.5% (8/106)

Note.^___^ Values are given as the mean ± standard deviation; values in parentheses represent ranges. MR, magnetic resonance

*Other than pretreatment with a GnRH agonist

^†^ Transformed to 0–100 [12]

^‡^ Type I: comparable to skeletal muscle; type II: lower than the myometrium and higher than the skeletal muscle; type III: equal to or higher than the myometrium, based on visual inspection [13]

### Screening MR Examination

To determine whether MR-HIFU ablation was feasible, screening MRI was performed with the patient in the prone position, using an MR scanner (1.5-Tesla; Achieva; Philips Healthcare, Best, The Netherlands) integrated into an MR-HIFU system (Sonalleve MR-HIFU Fibroid Therapy System; Philips Healthcare, Vantaa, Finland).

The screening MRI consisted of a routine T2-weighted image and perfusion MRI (Table 2). All examinations were evaluated by an operator (Y.S.K.) with 15 years of experience in MRI interpretation. On sagittal T2-weighted images, uterine size was measured from the uterine cervix to the farthest point of the uterus, usually at the fundus or the fibroid. Uterine configuration was assessed as either forward- or backward-bent according to the overall shape of the uterus and vagina in the pelvic cavity on sagittal images. The degree of bowel interposition was classified as none (approximated volume ratio of the target lesion affected by the interposed bowel loop <20%), partial (20–50%), or complete (>50%) [14].

**Table 2:** MR Imaging Parameters.

	Sequence	Repetition Time (ms)	Echo Time (ms)	Flip Angle (°)	Slice Thickness (mm)	Field of View (cm)	Matrix Size	Acquisition Duration/ Time Resolution (s)	Imaging Planes	Additional Information
Perfusion MR	*Fat-saturated T1-weighted FFE*	3.6	1.8	15 (pre); 15 (post)	6.0	25 × 25	208 × 206	3.0	Axial or coronal	100 dynamics (5 min)
Screening and pretreatment planning	*T2-weighted 3D TSE with DRIVE*	1000.0	135.0	90	2.5	25 × 25	168 × 120	200–220	Sagittal	SENSE 1.8
MR thermometry	*RF-spoiled segmented EPI*	37.0	19.5	19	7.0	40 × 25	160 × 100	2.9	Coronal, sagittal	121-binomial water-selective excitation
Immediate follow-up*	*Fat-saturated T1-weighted THRIVE*	6.6	3.2	10	2.5	28 × 28	240 × 240	170–190	Coronal	SENSE 1.5

DRIVE, driven equilibrium; EPI, echo planar imaging; FFE, fast field echo; MR, magnetic resonance; RF, radiofrequency; SENSE, sensitivity encoding; THRIVE, T1-weighted high resolution isotropic volume examination; TSE, turbo spin echo

* Gd-DOTA (0.1 mmol/kg; Dotarem, Guerbet, Aulnay-sous-Bois, France) was used for contrast enhancement.

If considered necessary, for instance, when the fibroid(s) had excessive size and/or vascularity, a gonadotropin releasing hormone (GnRH) agonist (leuprolide acetate or goserelin acetate; subcutaneous injection, repeated three times with an interval of 28 days) was administered before treatment [15, 16].

### Bowel Displacement Technique

**Fig 1:**
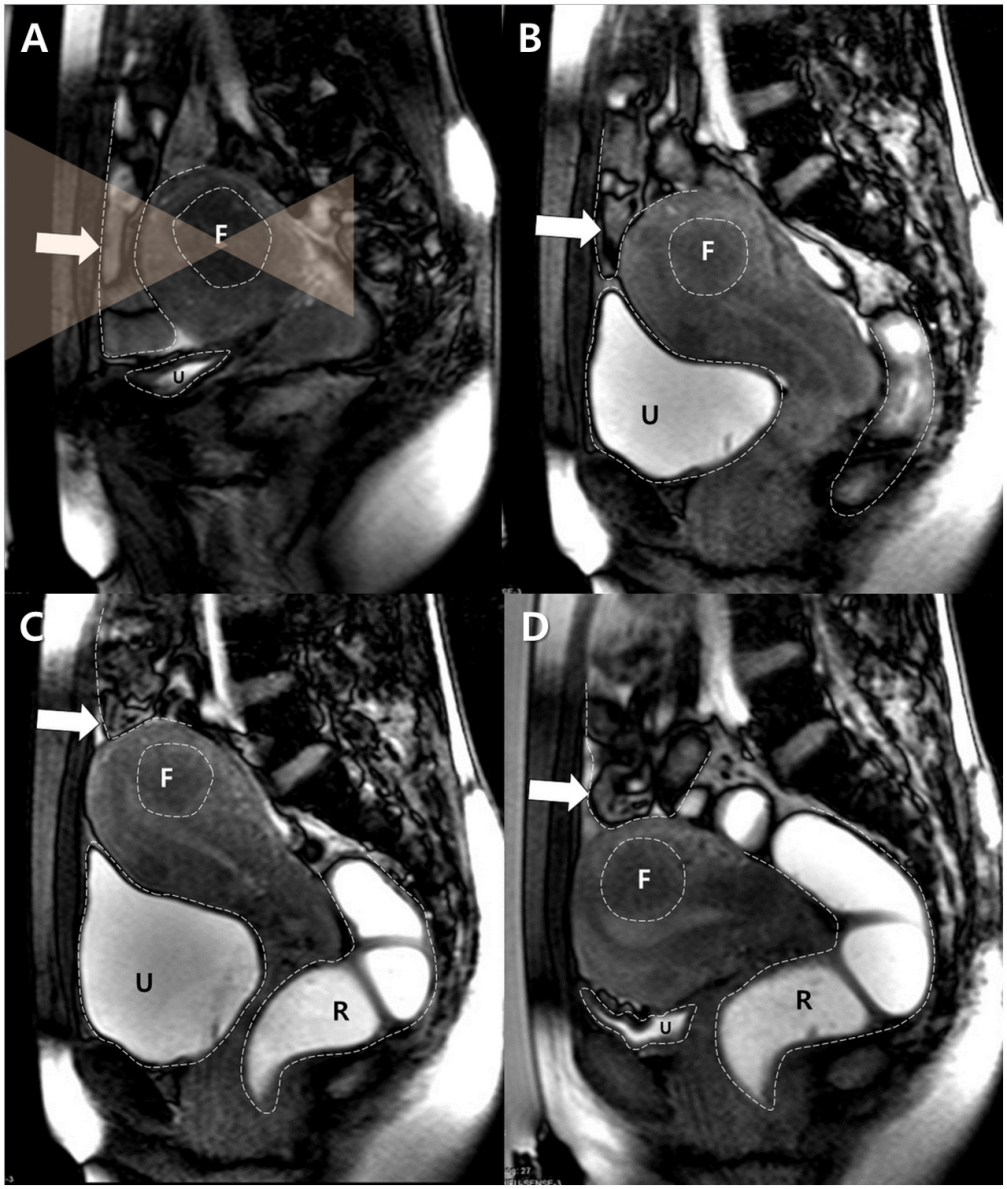
A typical example of the BRB maneuver attempted in the case of a 34 year-old woman with a fibroid in a forward-bent uterus. A. A sagittal survey scan showed that the bowel loops (arrow) were interposed between the uterus and the anterior abdominal wall. F and U indicate the target fibroid and the urinary bladder respectively. Dotted lines delineate the margins of each structure, and transparent orange triangles represent the planned HIFU beam path. B. After filling with 500 mL of saline, the urinary bladder (U) was distended and displaced the uterus cranially. However, the interposed bowel loops (arrow) were still in the anticipated sonication path. F indicates the target fibroid. Dotted lines delineate the margins of each structure. C. The rectum (R) was filled with 150 mL of gel. The distended rectum pushed the uterine cervix and the uterus antero-cranially, which displaced the bowel loops (arrow) out of the anticipated sonication path. F indicates the target fibroid and U indicates the urinary bladder. Dotted lines delineate the margins of each structure. D. The uterus descended after drainage of the urinary bladder (U), although the previously-interposed bowel loops (arrow) remained out of the anticipated sonication path. After a successful BRB maneuver, MR-HIFU ablation was performed. F indicates the target fibroid and R indicates the rectum. Dotted lines delineate the margins of each structure.

**Fig 2:**
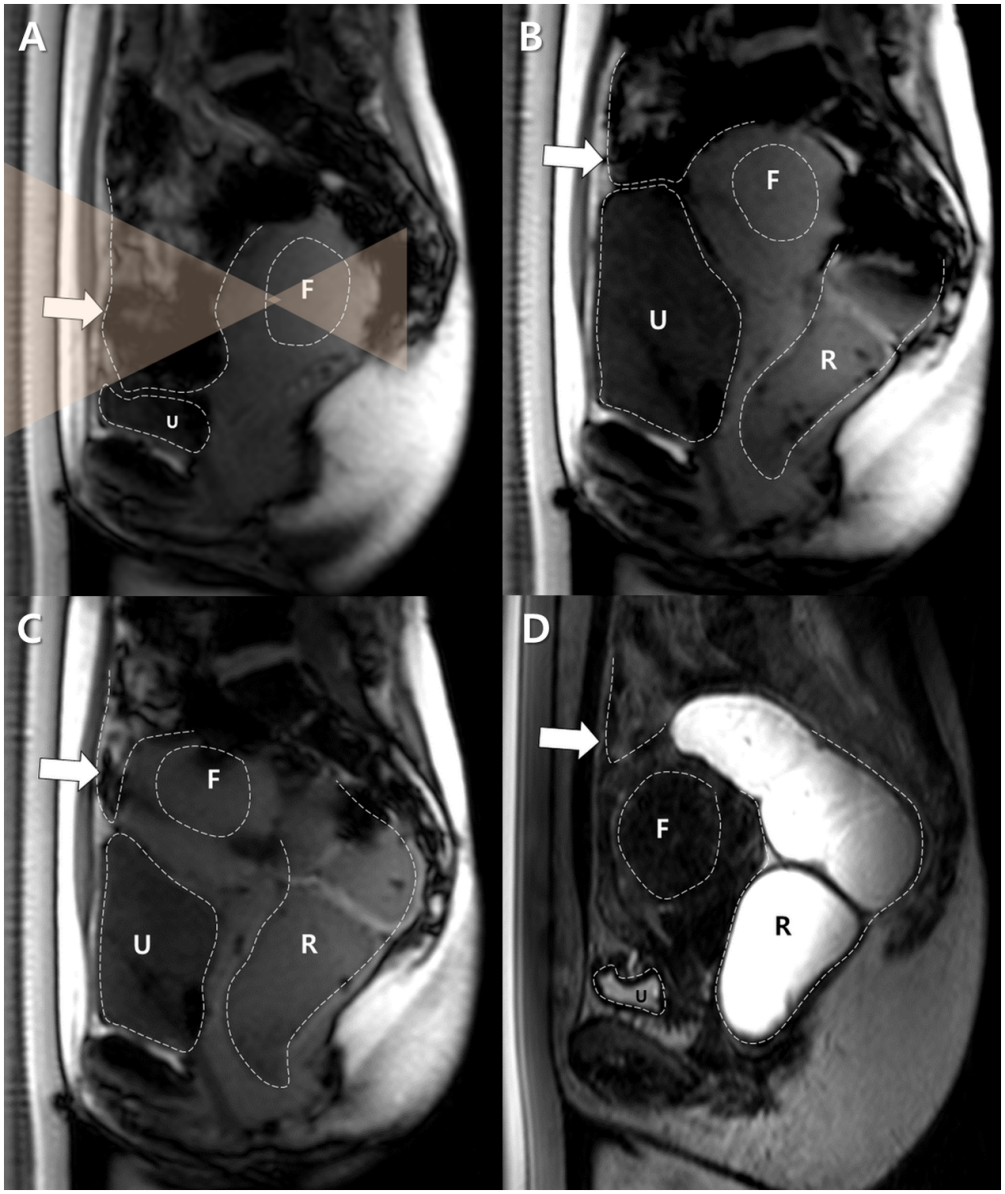
A successful BRB maneuver for a backward-bent uterus in a 43 year-old woman with a uterine fibroid. A. A sagittal survey scan revealed the backward-bent uterus located in the deep pelvic cavity and the interposed bowel loops. The size of the uterus was 90 mm in its largest dimension. F and U indicate the target fibroid and the urinary bladder, respectively. Dotted lines delineate the margins of each structure, and transparent orange triangles represent the planned HIFU beam path. B. The urinary bladder (U) was filled with 500 mL of saline and then the rectum (R) was filled with 100 mL of gel. The uterus was shifted antero-crainally. However, the bowel loops (arrow) continued to block the target fibroid (F). Dotted lines delineate the margins of each structure. C. The urinary bladder (U) was partially emptied by draining 100 mL of urine and the rectum (R) was filled further with 100 mL of gel. The target fibroid (F) was shifted anteriorly, but the bowel loops (arrow) were still in the anticipated sonication path. Dotted lines delineate the margins of each structure. D. After fully emptying the urinary bladder (U), the uterus was moved antero-caudally close to the abdominal wall, and the bowel loops (arrow) were displaced completely out of the sonication path. After a successful BRB maneuver, MR-HIFU ablation was performed. F indicates the target fibroid and R indicates the rectum. Dotted lines delineate the margins of each structure.

The BRB maneuver was used when the bowel loop was interposed between the anterior abdominal wall and the uterine fibroid(s) in the anticipated sonication path based on the survey scan, and the approximated volume ratio of the target fibroid(s) affected by the interposed bowel loop ≥ 20%. This technique consists of sequential urinary bladder filling with normal saline via a Foley catheter, rectal filling with ultrasound coupling gel via an enema syringe, and urinary bladder emptying (Figs 1 and 2). Bladder filling was performed by manually squeezing a plastic bag of saline connected to the side arm of a clamped Foley catheter, usually during preparation scans such as bubble detection or scar scans, and emptying was completed by de-clamping the catheter to allow natural drainage. Survey MR scans were intermittently performed during and after this technique to monitor internal organs. Each bladder filling used 300–500 mL of saline, and generally 100–150 mL of rectal gel was used and added if necessary. The amounts were determined based on survey scans and patient tolerance. If unsuccessful, bladder filling and emptying were repeated up to five times with or without additional rectal filling. If considered necessary, manual compression of the lower abdominal wall was performed by operator’s hand to prevent the bowel loops from descending during bladder emptying. The additional time taken for this technique was estimated as the difference in the average procedure preparation time (from arrival in the MR room to the first sonication) with or without the BRB maneuver.

This technique was first used in February 2011, and was then used routinely after the 5th time in November 2011 when the operator was convinced of its effectiveness. Pre- and post-BRB periods were based on this time point. In post-BRB period, bowel interposition was a sole reason for screening failure in no case.

### MR-HIFU System and Procedure

All MR-HIFU procedures used a clinical extracorporeal MR-HIFU system (Sonalleve MR-HIFU Fibroid Therapy System, V1 and V2 versions; Philips Healthcare, Vantaa, Finland), as previously described [17]. Patients fasted overnight, and a suppository-based laxative was used to prepare the bowel (Ducolax^®^ suppository; Boehringer Ingelheim, Ingelheim, Germany). A Foley catheter was inserted just before treatment. All MR-HIFU procedures were performed by a single interventional radiologist (Y.S.K.) with 11 years of experience in image-guided tumor ablation as of the beginning of patient accrual. The uterus was measured again using MRI on the treatment day. The procedure and medications were also described previously [17].

### Assessment of Procedure

BRB was considered a technical success if HIFU sonication was initiated after bladder emptying, but not if HIFU was delivered through the distended bladder without emptying (*i*.*e*., through-the-bladder sonication). BRB maneuver-related symptoms and complications were evaluated. The MR-HIFU procedure was considered to be a technical success if the target lesion(s) was treated according to the protocol and the treatment was continued as planned [18].

Contrast-enhanced MRI after intravenous Gd-DOTA administration (Table 2) was performed immediately after therapy to evaluate the non-perfused volume (NPV) (*i*.*e*., the fibroid area that lacked contrast enhancement). The NPV and fibroid volumes were quantified using an image-processing workstation (Virtual Place Advance Plus, Aze Co., Tokyo, Japan). A radiologist (Y.S.K) manually segmented NPVs and fibroids on immediate contrast-enhanced T1-weighted and pretreatment planning T2-weighted MRI, respectively. The NPV ratio was defined as the ratio of NPV to the fibroid volume (%).

### Statistical Analysis

Continuous variables are expressed as the mean ± standard deviation (range). Screening pass rates between pre- and post-BRB periods and rates of newly-developed bowel interposition on treatment day between groups with and without GnRH agonist pretreatment were compared using the chi-square or Fisher’s exact test. When the BRB maneuver was attempted (60 cases), risk factors for its technical failure (age, body mass index [BMI], history of full-term delivery, uterine configuration, uterine size on screening and treatment days, and GnRH agonist pretreatment) were assessed by logistic regression analysis using an enter method. In order to compare uterine size between cases with successful and unsuccessful BRB, Student’s T-test was used after verifying normal distribution of the residuals. A *P-*value <0.05 was considered statistically significant. Data were analyzed using IBM SPSS Statistics 22.0 (International Business Machines Corp., Armonk, NY).

* The first draft of this manuscript was written by one of authors (Y.S.K.).

## Results

### Screening MR Findings

In the screening MRI, the uterine size was 119.3 ± 25.7 (72–239) mm on sagittal images. The uterus was forward-bent in 319 cases and backward-bent in 56 cases. Bowel interposition was noted in 32.0% of cases (120/375) (complete, n = 96; partial, n = 24) (21.9% [70/319] of the forward-bent uterus; 89.3% [50/56] of the back-ward bent uterus).

The screening pass rates of pre- and post-BRB periods were 59.0% (98/166) and 71.7% (150/209) (*P* = 0.001), respectively. Bowel interposition was one of the reasons for failure in 60.3% (41/68) and 3.4% (2/59) of cases in the pre- and post-BRB period, respectively. Bowel interposition was the only reason for failure in 20.6% (14/68) and 0% (0/59), respectively. In bowel-interposed cases only, the corresponding pass rates were 14.6% (7/48) and 76.4 (55/72), respectively (*P* < 0.001). If we assumed that BRB maneuver was adopted during the pre-BRB period, 32 more cases might be eligible for MR-HIFU ablation, thus the screening pass rate could be 78.3% (130/166).

### Bowel Interposition in MR-HIFU Therapy

In 206 cases of MR-HIFU ablation, 72 cases (35.0%) showed bowel interposition (partial, n = 18; complete, n = 54). Among them, 27 cases (13.1%, 27/206) in which bowel interposition was not seen in screening MRI showed new bowel interposition on the treatment day. Conversely, in 9 cases (4.4%, 9/206), bowel interposition noted on the screening day was not apparent on the treatment day.

GnRH agonist pretreatment was performed for 33 patients (16.0%, 33/206). Of these, 21 did not show bowel interposition in screening MRI, although nine of 21 cases (42.9%) showed new bowel interposition on the treatment day, whereas new bowel interposition was seen in 18 out of 131 bowel-void cases (13.7%) in patients without GnRH agonist pretreatment (*P* = 0.001). Changes in uterine size were from 118.5 ± 28.5 (80–195) mm to 99.5 ± 22.1 (74–174) mm and from 122.9 ± 26.1 (78–239) mm to 122.9 ± 26.3 (68–239) mm, respectively, for groups with (n = 33) and without GnRH agonist pretreatment (n = 173).

### Bowel Displacement

**Fig 3:**
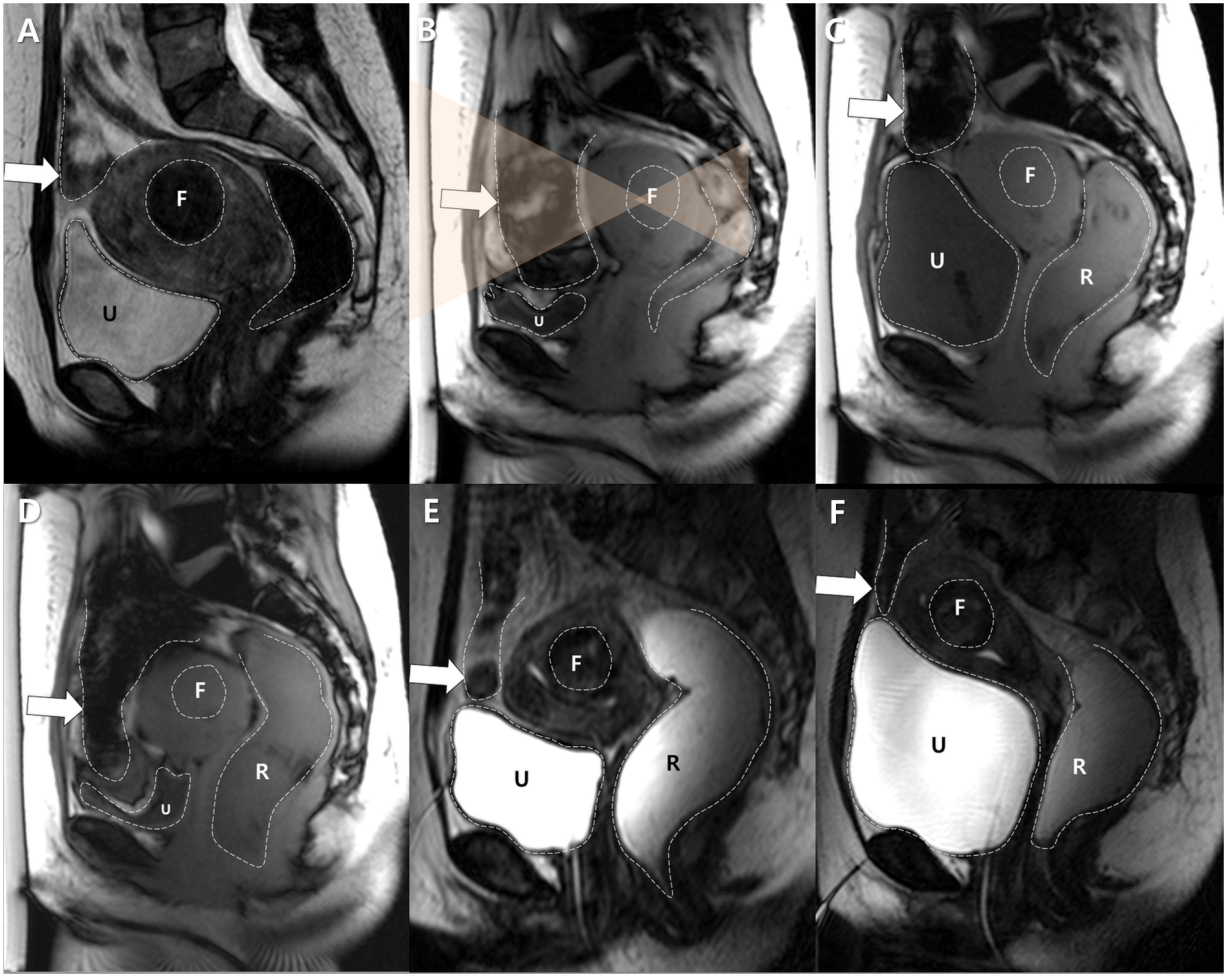
A case of technical failure of the BRB maneuver in a 42 year-old woman with a fibroid in a forward-bent uterus. The patient did not undergo GnRH agonist pretreatment. A. Sagittal image of screening MRI showed a uterine fibroid of the submucosal type (F). The uterus was bent forward and measured 108 mm in its largest dimension. The bowel loops were interposed. F indicates the target fibroid and U indicates the urine-filled urinary bladder. Dotted lines delineate the margins of each structure. B. On treatment day, a sagittal image from the survey scan revealed that more bowel loops (arrow) were interposed and the uterus shrank to 68 mm for an unknown reason. F and U indicate the target fibroid and the urinary bladder, respectively. Dotted lines delineate the margins of each structure, and transparent orange triangles represent the planned HIFU beam path. C. The urinary bladder (U) was filled, followed by the rectum (R). However, the bowels (arrow) were located within the anticipated sonication path, even after transducer angulation. Moreover, the target fibroid (F) was too deeply located to allow a safe and effective ablation. Dotted lines delineate the margins of each structure. D. After emptying the urinary bladder (U), the bowel loops (arrow) descended again completely blocking the target fibroid (F). R indicates the rectum. Dotted lines delineate the margins of each structure. E. The urinary bladder (U) was filled with saline (300 mL) again. However, there was still a bowel loop anterior to the uterus. Urinary bladder filling and emptying was repeated 5 times in this particular case. F and R indicate the target fibroid and the rectum, respectively. Dotted lines delineate the margins of each structure. F. Further filling of the urinary bladder (U) with saline (500 mL in total) and the rectum (R) with gel (350 mL in total) did not satisfactorily displace the interposed bowel loop (arrow). Therefore, the procedure was terminated without initiation of MR-HIFU sonication. Dotted lines delineate the margins of each structure.

In 72 cases with bowel interposition, the partially-interposed bowel loops were spontaneously displaced during ablation in two cases. In nine cases, an acoustic window was established by either bladder filling alone (n = 5; partial in three cases and complete in two cases) or rectal filling alone (n = 4; partial in all cases). In one case in the pre-BRB period, when we did not realize the effectiveness of the BRB maneuver, bowel interposition (complete in degree) could not be resolved by simple methods, and the procedure was therefore terminated without initiating sonication. In the remaining 60 cases (partial in nine cases and complete in 51 cases), the BRB maneuver was attempted. Bladder filling and emptying was repeated 1.7 ± 1.0 (1–5) times, and the amount of rectal gel used was 180 ± 86.5 (100–400) mL. In four cases, the maneuver succeeded after manual compression of the lower abdominal wall that was applied during the 3rd or 4th bladder emptying. The BRB maneuver established an acoustic window successfully in 49 cases, giving a technical success rate of 81.2% (49/60). In the remaining 11 cases (complete in all cases), the acoustic window was established after re-filling of the urinary bladder in 8 cases, therefore the treatment could be continued, however, because of the increased depth of the target lesion, smaller volumetric treatment cells (8 or 12 mm in axial diameter) needed to be used, lengthening the procedure time. In three cases, HIFU sonication could not be initiated because a safe acoustic window could not be established (Fig 3). The preparation time in cases without bowel interposition was 26.1 ± 7.0 (14–50) min, whilst it was 39.9 ± 12.4 (20–83) min in cases in which the BRB maneuver was used. Therefore, the mean additional time taken for the BRB maneuver was estimated to be 13.8 min. Overall, MR-HIFU ablation was successfully performed in 94.4% (68/72) of the bowel-interposed cases. There was no case in which the patient was not able to tolerate the BRB maneuver.

Among several factors analyzed, small uterine size on the treatment day was the only independently significant risk factor (B = 0.111, standard error = 0.046, *P* = 0.017) by multivariate analysis (Table 3). The uterine size on the treatment day in cases with a successful or unsuccessful BRB were 101.8 ± 15.2 (74–140) mm (n = 49) and 84.3 ± 10.9 (68–105) mm (n = 11), respectively (*P* = 0.001).

**Table 3:** Multivariate Analysis of Risk Factors for Failure of the BRB Maneuver.

	Estimate	Standard error	*p*-value	Odds ratio
*Intercept*	-2.203	6.850	0.748	0.110
*Age*	-0.029	0.129	0.823	0.971
*Body mass index*	-0.143	0.170	0.401	0.867
*History of full term delivery*	-0.196	1.196	0.870	0.822
Uterine configuration*	1.702	1.064	0.110	5.485
*Uterine size on screening day (cm)*	-0.038	0.036	0.292	0.963
*Uterine size on treatment day (cm)*	0.111	0.046	0.017^†^	1.117
*GnRH agonist pretreatment*	0.672	1.037	0.517	1.959

GnRH, gonadotropin releasing hormone

* Forward-bent vs. backward-bent

^†^Statistically significant, logistic regression analysis

Among three cases that finally failed to satisfactorily displace bowel loops even after trying through-the-bladder sonication, the uterus was forward-bent in two cases, and the patients underwent pretreatment with GnRH agonist in two cases. The uterine size on the treatment day was 68–89 mm, which was 32–49 mm smaller than on the screening day. Interestingly, and unusually, the uterus shrank by 40 mm in one patient who had not undergone hormone therapy (Fig 3).

Patients usually complained of varying strengths of desire to urinate when the urinary bladder was distended, and a temporary sensation of defecating lasting a few minutes immediately after filling the rectum, which were all tolerable. No BRB maneuver-related complications were identified.

### MR-HIFU Ablation

MR-HIFU ablation procedures were technically successful in 94.2% of cases (194/206). The reason for technical failure, which occurred in a total of 12 of 206 cases (5.8%), included a failure to displace the bowel (n = 4, previously described), and premature termination of the procedures either because a sufficient temperature rise was not achieved despite using the highest acoustic power output (n = 7), or due to a skin burn at the poorly-depilated area (n = 1). One to three doses of fentanyl hydrochloride (intravenous continuous infusion) were administered to control pain in 86.1% (174/202) of HIFU-insonated cases. Metoclopramide hydrochloride was used in four cases (4/202, 2.0%) to control nausea. The NPV of the treated fibroids in cases with technical success was 94.0 ± 128.2 (0–627.1) mL, and the NPV ratio was 73.9 ± 24.6 (0–100.0)%. NPVs and NPV ratios were 114.3±141.5 (0–627.1)mL and 73.8±24.0 (0–100.0)%, and 45.5±67.0 (1.2–391.7)mL and 75.0±25.0 (6.3–100.0)%, respectively for the cases without BRB maneuver and with BRB maneuver.

## Discussion

Several techniques have been suggested for moving interposed bowel loops out of the HIFU sonication path [9]. Urinary bladder filling with saline, which elevates the uterus and bowel cranially, could be useful for establishing a wider sonication window in cases with an anteverted/flexed uterus blocked at the antero-superior portion. Rectal filling can be performed with saline, ultrasound coupling gel, or an expandable balloon. Of these, ultrasound gel is preferred because of its relative stability and wide availability, and because it minimizes patient discomfort. Rectal distension pushes the uterus anteriorly or antero-cranially, which can displace the bowel loops peripherally. A convex gel pad can also be used to displace the interposed bowel loops peripherally by compressing the abdominal wall. However, this method may sacrifice focus-reachable depth, which could prevent the ablation of a deeply-located fibroid. The BRB maneuver used in our study, although more complicated than other simple techniques, allows an acoustic window to be safely and efficiently established. In addition, it maintains focus-reachable depth, and displaces the target toward the transducer, which increases the treatable portion of the fibroid and improves safety by increasing their distance from bony structures and sciatic nerves. In addition, it decreases the energy required owing to less acoustic attenuation which leads to less risks of skin burn and shorter cooling times.

In a BRB maneuver for a forward-bent (anteverted/flexed) uterus, bladder filling usually pushes the interposed bowel loops cranially (or more accurately, peripherally on a coronal view). The initial rectal filling (up to 100 mL) then pushes the uterus in an antero-cranial direction, compressing the posterior cervix along the distended mid to distal rectum. Further rectal filling (150–200 mL) may push the uterine body anteriorly and increase the tension on the distended urinary bladder and uterus, which is increased further by the effect of gravity on the uterine fibroid(s) in the prone position. Slow drainage of the urinary bladder then allows the uterine fundus to occupy the space previously filled by the bowel. Taken together, this explains why the size of the uterus influences the success of the BRB maneuver. A large uterus increases the chance of success because it fills the free peritoneal space in front of it. Manual compression of the abdominal wall also restricts the free peritoneal space temporarily, which may be important when the uterus is of borderline size. Further filling of the rectum causes a sensation of defecating, although this lasts only a few minutes, and migrates proximally to distend the sigmoid colon. In our cases, filling the rectum (probably the sigmoid colon as well) with a large amount of gel (up to 400 mL) was often beneficial as it widened the acoustic window by removing air inside the colon.

We initially used a BRB maneuver only in cases with a forward-bent uterus due to its mechanism of action. However, we then extended its use for patients with a backward-bent uterus, as it became apparent that the BRB maneuver was also effective in these cases. We found the uterus–vagina complex to be more flexible than expected as demonstrated in Fig 2. In our study, the BRB maneuver was used in 11 cases with a backward-bent uterus, and was technically successful in seven cases. In three cases, the procedures were successfully completed using through-the-bladder sonication, and only in one case was it not possible to initiate sonication. In cases with a forward-bent uterus, BRB maneuver was successful in 42 of 49 procedures, and in five cases, treatment was possible using through-the-bladder sonication. Finally, sonication was not able to be initiated in two cases.

We included the uterine size and configuration as potential risk factors for BRB failure for the above reasons. A history of full-term delivery was included because we thought that this might be associated with more flexibility, which is especially important in cases with a backward-bent uterus. GnRH agonist pretreatment is also known to induce fibroid shrinkage (*i*.*e*., a decrease in uterine size) [15, 16], and was therefore considered likely to affect the outcome of BRB maneuver. This was apparent in cases of BRB failure. However, it turned out not to be significant by multivariate analysis. It might be because the degree of volume shrinkage induced by GnRH agonist was not big enough to make BRB maneuver ineffective. Nonetheless, care must be taken by physicians when GnRH agonist is to be used.

Preliminary results for using the BRB maneuver in MR-HIFU ablation of uterine fibroids have been reported [10]. The study reported here included more cases (60 *vs*. 13), and we demonstrated that the maneuver could be used successfully in cases of a backward-bent uterus, discussed cases in which it failed (technical success rate, 81.2% in this study *vs*. 100.0% in the preliminary study), and showed that a small uterus was a risk factor for technical failure. Based on the findings reported here, the BRB maneuver can effectively resolve an interposed bowel, even if the uterus is backward-bent, although caution is needed if the uterus is small.

Multivariate analysis revealed that a small uterus on the treatment day was the only independent risk factor for BRB failure, although its size could not always be predicted based on screening, especially in patients undergoing GnRH agonist pretreatment. Based on our findings, we suggest the following modification of current clinical practice. For patients that do not need hormone therapy, the uterine size determined in the screening MR exam could be utilized. In patients who should receive hormone therapy before MR-HIFU ablation, uterine size on the treatment day could be predicted by subtracting 2 cm based on our result of uterine size changes by hormone therapy (*i*.*e*., from 118.5 mm to 99.5 mm in average). Taking into account the mean uterine size in BRB successful and unsuccessful cases, a ‘predicted’ uterine size of 9.0–9.5 cm on a sagittal image may act as a cut-off value to determine whether the BRB maneuver should be used.

When we retrospectively reviewed all of the screening MRI exams, we found that 39 out of 127 cases of screening failure (30.7%, mainly in the pre-BRB period) would in fact have been suitable for MR-HIFU based on our findings, increasing the overall screening pass rate from 66.1% (248/375) to 76.5% (287/375). At least 35 of the 39 cases could have been resolved using BRB maneuver. This eligibility rate is far higher than those from early studies (*i*.*e*., 16–25%) [5, 6]. We therefore suggest that bowel interposition should no longer prevent MR-HIFU ablation of the uterine fibroid, except when the uterus is small, because bowel interposition often varies between screening and treatment, and the technical success rate of bowel displacement is quite high. Moreover, the presence of a scar in the sonication path can be addressed using a scar patch [19], the beam shaping technology available in recent MR-HIFU systems [9], or even through-the-scar sonication for old and subtle scars [9]. Therefore, finally, the main reasons for procedure infeasibility seem to be a high T2 signal intensity and/or high perfusion of the fibroid [13, 20, 21], very large and/or numerous target lesion(s), a very deep location of the fibroid, or a very thick subcutaneous fat layer in the abdominal wall [11].

Our study has the following limitations. First, due to retrospective nature of our study, the bowel displacement techniques were performed based on clinical routines, thus could not be standardized, and a selection bias might be involved. Second, we simply divided the study period into pre-BRB and post-BRB periods for a methodological reason. However, experience levels of the operator might temporally differ even in the same period, which might bias the results. For instance, there were a few pre-BRB cases for which BRB maneuver was tried, especially those close to transition of the periods. We set a dividing point based on the operator’s conviction of technique effectiveness in pursuit of accuracy in post-BRB results. In fact, all bowel-interposed cases in post-BRB period passed screening and BRB maneuver was attempted.

In conclusion, highly effective bowel displacement techniques, including the BRB maneuver, allow the MR-HIFU ablation of uterine fibroids even in cases of bowel interposition, which should no longer be regarded as an exclusion criterion for this procedure. However, cases in which the patient has a small uterus should be approached cautiously.

## Supporting Information

S1 File Study data. Study data are provided in PDF format.
(PDF)
