# Supplementary material for: Magnetic Resonance Imaging-Guided High-Intensity Focused Ultrasound Ablation of Uterine Fibroids: Effect of Bowel Interposition on Procedure Feasibility and a Unique Bowel Displacement Technique
Source: PLoS One. 2016 May 17;11(5):e0155670. doi: 10.1371/journal.pone.0155670 (PMC4871469; doi:10.1371/journal.pone.0155670)
Supplement: S1 File — Study data are provided in PDF format. (PDF) [file pone.0155670.s001.pdf]

Pre/Post	Screening MR date	Age	BMI	Uterus size	Fibroid/adeno	Uterus loc	P+H Bowel	Planning bo	Scar Y/N	Pass/Fail	Reason for failure		Now?	MR-HIFU	HIFU bowel	Bowel method	Bowel success	BRB success	BRB Saline	BRB Gel	Tx day	Tech Success	No. of live birth	GnRH	Scar	Ut Size	Immediate Cx	Post ablation syn	MR room time	Prep Time
Pre	2010.11.16	50	23.2	72	F	P	Y	Y	N	F	BOWEL	Bowel	OK	N																
Pre	2010.11.16	45	28.78	148	F	A	N	N	N	P				Y	N						2010-11-24	Y	2	N	N	148	N	Y	250	50
Pre	2010.11.17	39	20.76	90	F	A	Y	P	Y	F	SCAR, BOWEL	Scar, bowel	OK	N																
Pre	2010.11.17	40	27.53	130	F	A	N	N	N	P				Y	N						2010-11-26	Y	0	N	N	130	Y	Y	260	45
Pre	2010.11.18	35	22.02	145	F	A	N	N	N	P				Y	N						2010-12-10	Y	0	N	N	145	Y		261	22
Pre	2010.11.18	43	18.11	125	F	A	N	N	N	P				N																
Pre	2010.11.18	50	25.97	152	F	A	N	N	N	P				Y	N						2010-12-06	Y	0	N	N	152	N		196	16
Pre	2010.11.22	42	25.4	78	F	A	Y	Y	N	F	BOWEL/DEPTH	Bowel, depth	NO	N																
Pre	2010.11.23	46	21.63	145	F	A	N	N	Y	P				Y	N						2010-12-15	Y	2	N	Y	145	Y		202	31
Pre	2010.11.23	41	20.74	115	F	A	N	N	N	P				N																
Pre	2010.11.23	42	22.85	119	F	A	N	N	N	P				N																
Pre	2010.11.23	48	22.43	125	F	A	N	N	Y	F	SCAR, SIZE	scar, load	OK	N																
Pre	2010.11.23	42	20.43	110	A	A	N	N	N	P				Y	N						2011-02-18	Y	2	N	Y	110	Y		195	26
Pre	2010.11.23	50	23.28	220	F	A	N	N	Y	F	SIZE, SCAR	scar, load	NO	N																
Pre	2010.11.24	47	21.75	115	F	A	N	N	N	F	T2PERF	T2perf	NO	N																
Pre	2010.11.24	46	26.12	119	F	A	N	N	N	P				N																
Pre	2010.11.29	46	26.49	105	A	A	N	N	N	F	DEPTH	Depth	OK	N																
Pre	2010.11.29	47		116	F	A	N	N	N	P				N																
Pre	2010.11.29	48	22.68	174	F	A	N	N	Y	P				Y	N						2010-12-07	Y	2	N	Y	174	N	Y	251	42
Pre	2010.11.30	42	24.1	104	F	P	N	N	Y	F	SIZE, DEPTH	load, depth	NO	N																
Pre	2010.12.01	43	26.66	132	F	A	N	N	N	P				Y	N						2010-12-16	Y	0	N	N	132	N		208	30
Pre	2010.12.01	40	19.06	117	F	A	N	N	N	P				Y	N						2010-12-13	Y	2	N	N	117	N		205	22
Pre	2010.12.01	45	23.18	110	F	A	Y	P	N	F	BOWEL	bowel	OK	N																
Pre	2010.12.01	46	23.32	137	F	A	N	N	N	P				Y	N						2010-12-22	Y	2	N	N	137	N	Y	239	27
Pre	2010.12.02	45	21.5	91	F	A	Y	N	N	P				Y	P	BLADDEF	Y				2011-01-14	Y	0	N	N	91	N		199	25
Pre	2010.12.02	44	22.3	86	F	P	Y	Y	N	F	BOWEL, DEPTH	bowel, depth	NO	N																
Pre	2010.12.03	36	20.53	105	A	A	Y	Y	Y	F	BOWEL, SCAR	scar, bowel	OK	N																
Pre	2010.12.05	42	22.68	154	F	A	N	N	Y	P				Y	N						2010-12-21	Y	2	N	Y	154	N		217	24
Pre	2010.12.07	37	24.19	113	F	A	N	N	N	F	DEG	deg	NO	N																
Pre	2010.12.07	49	19.4	106	F	A	Y	P	Y	F	SCAR, PERF, BO\	scar, bowel, T	OK	N																
Pre	2010.12.07	39	24.35	101	F	A	N	N	N	P				Y	N						2011-01-04	Y	2	N	N	101	Y		203	35
Pre	2010.12.08	41	21.17	206	F	A	N	N	N	P				Y	N						2010-12-24	Y	0	N	N	206	N		255	46
Pre	2010.12.09	45	22.66	144	F	A	N	N	N	P				Y	N						2011-01-06	Y	0	N	Y	144	N		216	27
Pre	2010.12.09	45	20.68	131	F	A	N	N	Y	F	DEG, SCAR	deg, scar	NO	N																
Pre	2010.12.09	43	19.83	125	F	A	N	N	Y	F	DEG	deg	NO	N																
Pre	2010.12.10	44	27.69	81	F	P	Y	N	N	P				Y	Y	BLADDEF	Y				2011-01-28	Y	2	N	N	81	N	Y	128	53
Pre	2010.12.12	37	21.95	102	F	A	Y	Y	N	F	BOWEL	bowel	OK	N																
Pre	2010.12.13	44	23.98	138	F	A	Y	N	N	P				Y	P	SPONTAI	Y				2011-01-10	Y	2	N	N	138	N		177	35
Pre	2010.12.14	40	21.38	110	F	P	Y	Y	N	F	BOWEL	bowel	OK	N																
Pre	2010.12.14	42	21.69	98	F	P	Y	Y	N	F	BOWEL	bowel	OK	N																

Pre	2010.12.16	46	27.82	97	F	P	Y	Y	N	F	BOWEL	bowel	OK	N														
Pre	2010.12.17	43	19.75	113	F	P	Y	Y	N	F	BOWEL	bowel	OK	N														
Pre	2010.12.17	43	22.74	125	F	A	N	N	N	P				N														
Pre	2010.12.19	48	24	113	A	A	Y	P	N	F	BOWEL	bowel	OK	N														
Pre	2010.12.19	49	22.36	136	F	A	Y	N	N	P				Y	Y	RECTUM	Y	100	2011-01-19	Y	2	N	N	136	Y	Y	200	58
Pre	2010.12.19	46	22.12	92	F	P	Y	Y	N	F	BOWEL	bowel	OK	N														
Pre	2010.12.20	45	20.35	130	F	A	N	N	N	P				Y	N				2010-12-29	Y	1	N	N	130	N	Y	175	42
Pre	2010.12.21	38	22.24	140	F	A	N	N	N	P				N														
Pre	2010.12.21	43	20.83	110	F	A	Y	Y	N	F	BOWEL	bowel	OK	N														
Pre	2010.12.21	50	26.31	88	F	P	Y	Y	N	F	BOWEL, DEPTH	Bowel, depth	NO	N														
Pre	2010.12.24	36	23.95	128	F	A	N	N	N	P				N														
Pre	2010.12.25	49	24.2	126	F	A	N	N	N	P				N														
Pre	2010.12.25	43	23.73	164	F	A	N	N	N	P				Y	N				2011-01-05	Y	0	N	N	164	N	Y	233	34
Pre	2010.12.25	47	26.57	128	FA	A	N	N	N	P				Y	N				2011-01-13	Y	2	N	N	128	N		232	30
Pre	2010.12.25	49	26.9	102	F	A	N	N	N	F	DEG	deg	NO	N														
Pre	2010.12.26	49	21.52	150	F	A	Y	N	N	P				Y	P	SPONTAI	Y		2011-02-09	Y	2	N	N	150	N		145	21
Pre	2010.12.26	43	20.13	108	F	A	Y	Y	N	F	BOWEL	bowel	OK	N														
Pre	2010.12.27	42	23.12	138	A	A	N	N	Y	P				Y	N				2011-01-26	Y	0	N	Y	138	N		187	22
Pre	2010.12.27	44	24.95	98	F	A	Y	Y	Y	F	BOWEL	bowel	OK	N														
Pre	2010.12.28	30	22.65	105	A	A	N	N	N	P				Y	N				2011-01-18	Y	0	N	N	105	N		161	33
Pre	2010.12.29	39	29.21	139	F	A	N	N	Y	P				Y	N				2011-01-17	Y	2	N	Y	139	Y		176	35
Pre	2011.01.01	36	21.15	120	F	A	Y	Y	Y	F	BOWEL, SCAR	scar, bowel	OK	N														
Pre	2011.01.01	45	23.89	115	F	A	N	N	N	P				Y	N				2011-01-24	Y	2	N	N	115	Y		227	26
Pre	2011.01.02	47	21.3	166	F	A	N	N	N	P				Y	N				2011-01-12	Y	2	N	Y	166	N	Y	244	25
Pre	2011.01.04	50	23.49	144	F	A	N	N	N	P				Y	N				2011-02-11	Y	2	N	N	144	N		224	18
Pre	2011.01.08	48	23.89	157	F	A	N	N	Y	F	DEPTH	Depth	NO	N														
Pre	2011.01.08	50	20.22	179	F	A	N	N	Y	P				N														
Pre	2011.01.14	48	22.6	143	A	A	N	N	N	P				Y	N				2011-01-25	Y	0	N	Y	143	N		203	32
Pre	2011.01.15	46	21.78	107	F	A	Y	Y	Y	F	DEPTH, BOWEL	bowel, depth	NO	N														
Pre	2011.01.15	51	21.24	138	F	P	N	N	N	P				N														
Pre	2011.01.16	35	22.18	93	F	A	N	N	N	P				N														
Pre	2011.01.25	46		163	F	A	N	N	N	F	T2PERF	T2perf	NO	N														
Pre	2011.01.29	43	22.18	155	F	A	N	N	N	P				Y	N				2011-03-04	Y	0	N	N	155	N		250	22
Pre	2011.01.29	31	23.73	115	F	A	N	N	N	P				N														
Pre	2011.01.29	48	22.64	127	F	A	N	N	Y	F	TOO HIGH, SCAI	too high, scar	NO	N														
Pre	2011.01.30	47	20.84	120	F	A	Y	P	N	F	BOWEL, DEPTH	bowel, depth	OK	N														
Pre	2011.01.30	47	22.51	157	F	A	N	N	N	P				Y	N				2011-02-25	Y	2	N	N	157	N	Y	205	25
Pre	2011.01.30	42	22.89	125	F	A	N	N	N	P				Y	N				2011-02-21	Y	0	N	N	125	N		221	23
Pre	2011.01.30	42	25.51	106	F	A	N	N	N	P				N														
Pre	2011.01.30	44	20.82	110	F	A	N	N	N	P				Y	N				2011-02-23	N	1	N	N	110	N		320	
Pre	2011.02.12	33	20.48	116	F	A	N	N	Y	F	DEG	deg	NO	N														
Pre	2011.02.16	38	18.96	145	F	A	N	N	N	P				Y	N				2011-03-11	Y	2	N	N	145	N		182	23
Pre	2011.02.16	47	22.6	119	F	A	N	N	N	P				Y	N				2011-02-28	Y	1	N	N	119	N	Y	200	34

Pre	2011.02.19	49	22.62	125	F	A	Y	Y	N	F	BOWEL	bowel	OK	N															
Pre	2011.02.20	45	22.09	141	F	A	N	N	N	F	SIZE, DEPTH	load, depth	NO	N															
Pre	2011.02.20	39	30.4	120	F	A	N	N	Y	F	SCAR	scar	OK	N															
Pre	2011.02.22	43		115	F	A	N	N	N	F	DEPTH	Depth	NO	N															
Pre	2011.02.24	43	21.01	86	F	P	Y	Y	N	F	DEPTH, BOWEL	Bowel, depth	NO	N															
Pre	2011.02.24	50	24.64	122	F	A	N	N	N	P				Y	N					2011-03-07	Y	2	N	N	122	N		163	20
Pre	2011.02.26	46	21.43	98	F	P	Y	Y	N	F	BOWEL	bowel	OK	N															
Pre	2011.02.27	44	19.81	184	F	A	N	N	Y	P				Y	N					2011-03-09	Y	2	N	Y	184	N		271	22
Pre	2011.02.27	47	21.8	105	F	P	Y	Y	N	F	BOWEL	bowel	OK	N															
Pre	2011.02.27	37	23.23	139	F	A	Y	N	N	P				Y	Y	BRB	Y	Y	100	2011-03-18	Y	2	N	N	139	N		233	52
Pre	2011.03.02	42	25.27	144	FA	A	N	N	N	P				Y	N					2011-03-25	Y	1	N	N	144	N		205	19
Pre	2011.03.04	43	23.12	96	F	A	Y	Y	Y	F	BOWEL, DEPTH	Bowel, depth	OK	N															
Pre	2011.03.10	41	21.26	134	A	A	N	N	N	P				Y	N					2011-03-16	Y	2	N	Y	134	N		233	30
Pre	2011.03.16	27	21.97	85	A	A	N	N	N	P				N															
Pre	2011.03.17	51	22.86	122	F	A	N	N	N	P				N															
Pre	2011.03.23	43	17.37	92	F	A	N	N	N	P				N															
Pre	2011.03.23	35		108	FA	A	N	N	Y	F	DEPTH, SCAR	depth, scar	NO	N															
Pre	2011.03.24	35	22.58	107	F	A	N	N	N	P				N															
Pre	2011.03.26	51	21.91	116	F	A	N	N	N	P				Y	N					2011-04-20	Y	3	N	N	116	Y		190	20
Pre	2011.03.30	50	26.09	104	F	A	Y	P	N	P				N						2011-05-02	N	2	N	Y		N		150	
Pre	2011.04.06	47	18.69	128	F	A	N	N	N	P				Y	N					2011-04-22	Y	2	N	N	128	N	Y	198	23
Pre	2011.04.12	39	20.37	119	F	A	N	N	N	P				Y	N					2011-05-09	Y	2	N	N	119	N		197	45
Pre	2011.04.19	47		107	F	A	N	N	Y	P				N															
Pre	2011.04.21	33	18.75	130	F	A	N	N	N	F	DEPTH	depth	NO	N															
Pre	2011.04.21	43		116	FA	A	Y	Y	Y	F	DEPTH, BOWEL	bowel, depth	NO	N															
Pre	2011.04.25	28	20.01	128	F	A	N	N	N	P				N															
Pre	2011.04.27	44	25.94	131	F	A	N	N	N	P				Y	N					2011-05-11	Y	1	N	N	131	Y		265	20
Pre	2011.04.28	41	21.36	137	F	A	N	N	N	F	SIZE, DEG, DEPT	deg, load, de|	NO	N															
Pre	2011.05.06	43	17.47	89	F	A	Y	P	N	P				N															
Pre	2011.05.09	25	22.99	113	F	A	N	N	Y	P				Y	N					2011-05-30	N	0	N	Y	113	Y		162	32
Pre	2011.05.10	29	22.78	89	F	A	N	N	N	F	T2PERF	T2perf	NO	N															
Pre	2011.05.10	32	20.36	89	F	A	Y	Y	N	F	BOWEL	bowel	OK	N															
Pre	2011.05.12	50	27.48	190	F	A	N	N	N	F	SIZE, DEPTH	load, depth	NO	N															
Pre	2011.05.17	46	20.54	82	F	P	Y	Y	Y	F	BOWEL	bowel	OK	N															
Pre	2011.05.20	44	27.22	173	F	A	N	N	N	P				Y	N					2011-06-29	Y	0	N	N	173			329	28
Pre	2011.06.03	37	21.79	156	F	A	N	N	N	P				Y	N					2011-06-22	Y	0	N	N	156	N		243	18
Pre	2011.06.07	32	19.45	92	A	P	Y	Y	Y	F	DEPTH, BOWEL	bowel, depth	NO	N															
Pre	2011.06.08	42	20.7	172	F	A	N	N	N	P				Y	N					2011-07-06	Y	0	N	N	172	N		300	20
Pre	2011.06.16	39	21.56	104	F	A	Y	N	N	P				Y	P	RECTUM	Y		100	2011-10-26	Y	2	Y	N	82	Y		220	26
Pre	2011.06.21	43	21.07	105	A	P	Y	Y	N	F	BOWEL	bowel	OK	N															
Pre	2011.06.23	39	19.1	101	F	P	Y	Y	N	F	BOWEL	bowel	OK	N															
Pre	2011.06.26	38	16.9	150	F	A	N	N	N	P				Y	N					2011-07-20	Y	0	N	N	150	N		227	31
Pre	2011.07.05	41	26.09	101	F	A	Y	Y	Y	F	BOWEL	bowel	OK	N															

Pre	2011.07.16	52	25	146	F	A	N	N	N	P				Y	N					2011-10-12	Y	3	Y	N	92	Y		230	31
Pre	2011.07.25	42	24.85	142	F	A	N	N	N	P				Y	N					2011-08-03	Y	2	N	N	142	N		273	22
Pre	2011.07.30	35	20.46	119	F	A	N	N	N	P				N															
Pre	2011.07.31	37	20.79	96	F	A	Y	N	N	P				Y	P	BLADDEF	Y			2011-08-17	Y	0	Y	N	113	N		250	26
Pre	2011.08.04	42	26.07	79	A	P	Y	Y	N	F	DEPTH, BOWEL	Bowel, depth	NO	N															
Pre	2011.08.05	39	26.21	139	F	A	N	N	N	P				Y	N					2011-08-26	Y	0	N	N	139	N		255	18
Pre	2011.08.07	35	20.54	158	F	A	N	N	N	F	SIZE, T2PERF	load, t2perf	NO	N															
Pre	2011.08.09	45	22.51	116	F	A	N	N	N	P				Y	N					2011-09-16	Y	3	N	N	116	N		202	32
Pre	2011.09.18	45	22.29	140	F	A	N	N	Y	P				Y	N					2011-10-14	Y	2	N	Y	140	Y		245	35
Pre	2011.09.20	50	20.31	103	F	P	Y	Y	N	F	BOWEL	bowel	OK	N															
Pre	2011.09.21	52	23.39	102	F	A	Y	P	N	P				Y	Y	BRB	Y	Y	100	2011-09-23	Y	2	N	N	96	N		204	33
Pre	2011.09.27	50	25.91	181	F	A	N	N	N	P				Y	N					2011-10-21	Y	2	N	N	181	N		255	24
Pre	2011.09.28	46	22.72	110	F	A	N	N	Y	F	T2PERF	T2perf	NO	N															
Pre	2011.10.06	40	19.28	162	F	A	N	N	Y	P				Y	N					2011-10-19	Y	1	N	Y	162	Y		328	26
Pre	2011.10.10	51	24.03	195	F	A	N	N	N	P				Y	N					2011-11-02	Y	0	N	N	195	N		275	35
Pre	2011.10.27	37	20.82	78	F	A	Y	N	N	P				Y	Y	BRB	Y	Y	100	2011-12-16	Y	2	N	Y	100	N		180	55
Pre	2011.10.28	47	25.67	130	F	A	N	N	N	P				N															
Pre	2011.11.07	47	20.83	140	F	A	Y	N	N	P				Y	Y	BLADDEF	Y			2012-03-07	N	1	Y	N	94			110	35
Pre	2011.11.17	47	21.82	118	F	A	Y	Y	N	F	BOWEL	bowel	OK	N															
Pre	2011.12.01	37	19	122	F	A	N	N	N	P				Y	N					2011-12-21	Y	0	N	N	122	N		217	28
Pre	2011.12.08	39	20.55	97	F	P	Y	Y	N	F	BOWEL	bowel	OK	N															
Pre	2011.12.23	43	21.72	127	F	A	N	N	Y	P				Y	N					2012-02-22	Y	0	N	Y	127	N		230	36
Pre	2011.12.24	45	23.34	139	F	A	N	N	N	P				Y	N					2012-01-20	Y	2	N	Y	139	N		232	30
Pre	2011.12.26	44	27.1	141	FA	A	N	N	N	F	DEPTH, T2PERF	depth, t2perf	NO	N															
Pre	2012.01.02	42	18.35	96	A	P	Y	Y	N	F	DEPTH, BOWEL	bowel, depth	NO	N															
Pre	2012.01.03	39	20.14	106	F	A	Y	P	N	P				Y	Y	BRB	Y	Y	100	2012-01-04	Y	0	N	N	112	N		228	44
Pre	2012.01.04	41	20.52	100	F	A	N	N	N	P				N															
Pre	2012.01.05	47	25.89	130	F	A	N	N	Y	P				Y	N					2012-03-28	Y	1	Y	Y	112	N		155	25
Pre	2012.01.06	48	21.7	123	F	A	N	N	N	P				Y	N					2012-01-27	Y	2	Y	N	119	N		210	33
Pre	2012.01.11	39	26.19	137	F	A	N	N	N	F	SIZE, T2PERF	load, t2perf	NO	N															
Pre	2012.01.18	37	23.65	92	F	A	Y	Y	N	P				Y	Y	BRB	Y	Y	200	2012-02-15	Y	1	N	N	95	N		158	50
Pre	2012.02.01	51	26.01	132	F	A	N	N	N	P				Y	N					2012-02-24	Y	2	N	N	132	N	N	160	22
Pre	2012.02.09	50	25.95	104	F	A	N	N	Y	P				Y	N					2012-02-10	Y	2	N	Y	104	N		130	35
Pre	2012.02.11	40	19.54	108	F	A	Y	P	N	F	BOWEL	bowel	OK	N															
Pre	2012.02.14	29	28.76	134	F	A	N	N	N	F	FAT+SIZE, DEPTI	fat, load, dep	NO	N															
Pre	2012.02.15	40	20.95	103	F	A	Y	Y	N	P				Y	Y	THRU BL	Y	N	150	2012-03-30	Y	0	Y	N	95	Y		185	65
Pre	2012.02.16	44	24.79	92	FA	A	N	N	N	F	T2PERF	T2perf	NO	N															
Pre	2012.02.17	44	22.1	96	F	A	Y	P	N	P				N															
Pre	2012.02.23	42	17.54	96	A	P	Y	Y	N	F	BOWEL	bowel	OK	N															
Pre	2012.02.23	44	22.86	109	F	P	Y	Y	N	F	BOWEL	bowel	OK	N															
Post	2012.03.07	39	19.31	114	F	A	Y	N	N	P				Y	P	BRB	Y	Y	100	2012-04-06	Y	0	N	N	107	N	N	185	35
Post	2012.03.09	44	25.88	86	F	A	Y	Y	N	P				Y	Y	BRB	Y	Y	150	2012-03-16	Y	0	N	N	84	N		181	36
Post	2012.03.16	44	23.83	136	F	A	Y	P	N	F	DEPTH	depth	NO	N															

Post	2012.03.30	48	21.59	134	F	A	N	N	N	F	DEPTH, SIZE	load, depth	NO	N																
Post	2011.09.28	42	24.75	122	F	A	N	N	N	P				Y	N						2012-04-27	Y	0	N	N	122	N	N	182	25
Post	2012.04.12	43	17.84	130	F	A	Y	N	N	P				Y	P	BRB	Y	Y		100	2012-05-04	Y	0	N	N	123	N	N	294	33
Post	2012.04.27	49	21.18	131	F	A	N	N	N	F	T2PERF+SIZE	load, t2perf	NO	N																
Post	2012.05.11	34	21.47	122	F	A	Y	N	N	P				Y	P	RECTUM	Y			100	2012-05-23	Y	0	N	N	122	N	N	221	34
Post	2012.05.29	43	20.5	116	F	A	N	N	N	P				Y	N						2012-07-11	Y	0	N	N	116	N	N	200	30
Post	2012.06.04	39	19.82	116	F	A	N	N	N	P				Y	N						2012-06-20	Y	0	Y	N	98	N	N	228	22
Post	2012.06.10	41	19.91	134	FA	A	N	N	N	F	ADENO+SIZE	load	NO	N																
Post	2012.06.20	50	20.82	78	F	P	Y	Y	N	F	DEPTH, BOWEL	Bowel, depth	NO	N																
Post	2012.06.27	46	19.54	119	F	A	N	N	Y	P				Y	N						2012-07-13	Y	3	N	Y	119	N	N	180	26
Post	2012.06.29	45	24.55	140	F	A	N	N	Y	F	T2PERF+SIZE	load, t2perf	OK	N																
Post	2012.07.02	49	23.99	105	F	A	N	N	Y	F	T2PERF	t2perf	NO	N																
Post	2012.07.04	49	20.87	119	F	A	N	N	Y	F	SIZE, DEPTH	load, depth	NO	N																
Post	2012.07.05	35	27.85	91	F	A	N	N	Y	F	FAT, T2PERF	fat, t2perf	NO	N																
Post	2012.07.06	48	19.26	106	F	A	Y	P	N	P				Y	N						2012-07-24	Y	2	N	N	106	N	Y	244	20
Post	2012.07.19	49	23.28	115	A	A	N	N	N	F	ADENO+SIZE	load	NO	N																
Post	2012.07.20	53	23.16	106	F	A	Y	Y	N	P				Y	Y	BRB	Y	Y	300	100	2012-09-26	Y	2	N	N	110	Y	N	206	46
Post	2012.07.30	33	19.24	112	F	A	N	N	N	F	T2PERF	t2perf	NO	N																
Post	2012.08.03	48	23.77	124	F	A	Y	N	N	P				Y	Y	BRB	Y	Y		150	2012-08-22	Y	2	N	N	117	N	N	215	27
Post	2012.08.03	45	21.75	130	F	A	N	N	Y	P				Y	N						2012-08-31	Y	2	N	Y	130	N	N	211	28
Post	2012.08.06	38	20.84	103	F	A	Y	Y	N	P				Y	Y	BRB	Y	Y	400	100	2012-08-10	Y	2	N	N	105	N	N	177	35
Post	2012.08.06	50	22.62	124	F	A	N	N	N	F	T2PERF+SIZE	load, t2perf	NO	N																
Post	2012.10.22	45	23.35	130	F	A	N	N	N	P				Y	N						2012-11-16	Y	2	N	N	130	N	N	184	29
Post	2012.11.23	31	24	125	F	A	N	N	N	P				Y	N						2012-12-28	Y	0	N	N	125	N	N	245	28
Post	2012.12.05	40	21.97	107	F	P	Y	Y	N	F	DEPTH	depth	NO	N																
Post	2012.12.18	46	27.92	180	F	A	N	N	N	P				Y	N						2012-12-21	Y	0	N	N	180	N	N	270	20
Post	2012.12.24	45	21.22	86	F	A	N	N	Y	P				Y	N						2012-12-26	Y	1	N	Y	86	N	N	200	38
Post	2013.01.02	34	21.86	86	A	P	Y	Y	N	F	DEPTH, BOWEL	bowel, depth	NO	N																
Post	2013.01.10	55	23.18	129	F	A	N	N	N	P				Y	N						2013-01-23	Y	0	N	N	129	N	N	227	26
Post	2013.01.10	43	23.48	101	F	A	N	N	N	P				N																
Post	2013.01.11	41	20.86	163	F	A	N	N	N	P				Y	N						2013-02-01	Y	0	N	N	163	N	N	250	28
Post	2013.01.11	45	22.27	103	F	A	Y	Y	N	F	T2PERF	t2perf	NO	N																
Post	2013.01.11	46	21.66	130	F	A	N	N	N	P				Y	N						2013-02-27	Y	1	N	N	130	N	Y	285	30
Post	2013.01.23	37	21.02	102	F	A	N	N	Y	P				Y	N						2013-02-06	Y	2	N	Y	102	Y	N	300	39
Post	2013.01.30	45	22.04	94	F	P	Y	Y	N	P				N																
Post	2013.02.05	46	21.69	93	F	P	Y	Y	N	F	DEPTH	depth	NO	N																
Post	2013.02.12	49	23.82	145	A	A	N	N	Y	F	ADENO+SIZE	load	NO	N																
Post	2013.02.25	42	21.35	147	F	A	N	N	Y	F	T2PERF+SIZE	load, t2perf	NO	N																
Post	2013.02.28	46	21.94	114	F	A	N	N	N	P				Y	N						2013-04-03	Y	0	N	N	114	N	N	225	23
Post	2013.03.11	48	23.07	157	FA	A	N	N	N	P				Y	N						2013-04-17	Y	3	N	N	157	N	N	270	21
Post	2013.03.13	43	24.64	169	F	A	N	N	N	P				Y	N						2013-08-30	Y	0	N	N	169	N	N	279	22
Post	2013.03.15	32	24.38	74	F	A	Y	Y	N	F	DEG	deg	NO	N																
Post	2013.03.18	46	18.13	83	F	P	Y	Y	N	P				N																

Post	2013.03.19	49	22.22	111	F	A	Y	P	N	P				Y	P	BRB	Y	Y		100	2013-04-05	Y	2	N	N	101	N	N	260	38
Post	2013.03.22	46	31.7	96	F	A	N	N	N	P				N																
Post	2013.04.03	46	27.93	99	F	P	N	N	N	F	DEPTH	depth	OK	N																
Post	2013.04.17	37	19.95	147	F	A	N	N	N	F	T2PERF+SIZE	load, t2perf	NO	N																
Post	2013.04.19	45	23.33	91	F	A	Y	Y	N	P				Y	N						2013-07-05	Y	3	N	N	91	Y	N	230	
Post	2013.04.30	53	24.74	167	F	A	N	N	N	F	T2PERF+SIZE	load, t2perf	NO	N																
Post	2013.05.08	39	25.56	152	F	A	N	N	Y	P				Y	N						2013-06-21	Y	2	N	Y	152	Y	Y	227	32
Post	2013.05.16	41	32.01	107	A	A	N	N	N	F	DEPTH	depth	OK	N																
Post	2013.05.22	38	20.9	91	F	A	Y	N	N	P				Y	Y	BRB	Y	Y	500	300	2013-06-12	Y	0	N	N	100	N	Y	205	50
Post	2013.05.31	45	24.28	108	F	A	Y	Y	N	P				Y	Y	BRB	N	N		350	2013-06-14	N	1	N	Y	68			150	
Post	2013.06.07	46	20.75	80	F	A	Y	N	N	P				Y	Y	BRB+ma:	Y	Y		200	2013-08-07	Y	2	N	N	85	N	N	165	55
Post	2013.06.05	43	27.53	140	F	A	N	N	N	P				Y	N						2013-07-12	Y	0	N	N	140	N	N	255	20
Post	2013.06.10	49	23.03	111	FA	A	Y	N	N	P				Y	P	RECTUM	Y			100	2013-07-03	Y	1	N	N	111	N	Y	150	13
Post	2013.06.10	39	22.44	87	FA	A	Y	Y	Y	P				Y	Y	BRB+ma:	Y	Y		150	2013-08-09	Y	0	Y	Y	88	Y	N	272	83
Post	2013.06.11	38	22.18	116	F	A	Y	P	N	P				Y	N						2013-07-26	Y	0	N	N	116	Y	N	286	26
Post	2013.06.21	42	20.32	100	F	A	N	N	N	P				N																
Post	2013.06.24	46	21.63	115	F	A	Y	Y	N	P				Y	Y	BRB	Y	Y		300	2013-07-24	Y	2	N	N	112	N	N	172	33
Post	2013.06.27	55	23.04	124	F	A	N	N	N	F	DEPTH	depth	OK	N																
Post	2013.07.08	53	24.2	107	F	A	N	N	Y	P				Y	N						2013-07-31	Y	2	N	N	107	N	N	280	18
Post	2013.07.11	40	21.61	98	A	A	N	N	Y	P				Y	N						2013-08-02	Y	2	N	Y	98	N	N	172	28
Post	2013.07.11	43	26.01	100	F	A	N	N	N	P				Y	N						2013-07-19	Y	0	N	N	100	Y	N	173	23
Post	2013.07.17	43	18.8	87	F	A	Y	N	Y	P				Y	Y	BRB	Y	Y		100	2013-08-16	Y	1	Y	Y	81	N	N	275	51
Post	2013.07.23	35	22.35	109	A	A	N	N	N	F	T2PERF+SIZE	load, t2perf	NO	N																
Post	2013.07.24	39	19.29	106	F	A	Y	N	N	P				Y	Y	BRB	Y	Y		200	2013-12-11	Y	0	N	N	130	N	N	233	48
Post	2013.07.26	45	23.51	120	F	A	N	N	Y	P				Y	N						2013-09-04	Y	1	N	N	120	N	N	257	27
Post	2013.08.01	41	20.57	130	F	A	N	N	N	P				Y	N						2013-11-08	Y	0	N	N	130	N	N	315	20
Post	2013.08.05	46	20.7	81	F	P	Y	Y	Y	P				Y	Y	BRB	Y	Y		150	2013-08-23	Y	1	N	Y	83	N	N	158	43
Post	2013.08.06	45	19.65	96	F	A	Y	Y	N	P				Y	Y	THRU BL	Y	N		250	2013-08-21	Y	2	N	N	96	N	N	273	59
Post	2013.08.12	44	19.63	100	F	A	N	N	N	P				Y	N						2013-10-25	Y	2	N	N	100	N	N	257	32
Post	2013.08.16	45	19.96	117	F	A	Y	P	N	P				Y	P	BLADDEF	Y				2013-09-06	Y	1	N	N	117	Y	N	265	24
Post	2013.08.23	38	26.11	105	F	A	N	N	N	P				Y	N						2013-09-11	Y	0	N	N	105	N	N	255	39
Post	2013.08.27	37		136	F	A	N	N	N	F	DEG	deg	NO	N																
Post	2013.09.02	28	19.1	92	A	A	Y	N	N	P				Y	Y	BRB	Y	Y		100	2013-09-25	Y	0	N	N	98	N	N		33
Post	2013.09.03	49		82	F	P	Y	Y	N	F	DEPTH	depth	NO	N																
Post	2013.09.04	49		131	F	A	N	N	Y	F	T2PERF+SIZE	load, t2perf	NO	N																
Post	2013.09.06	42	22.41	112	A	P	Y	Y	N	P				Y	Y	BRB	Y	Y		200	2013-10-04	Y	1	N	N	111	Y	N	280	35
Post	2013.09.06	41	22.31	99	F	A	N	N	N	P				Y	N						2013-10-23	Y	2	N	N	99	N	N	170	23
Post	2013.09.09	46		133	F	A	N	N	N	F	DEG	deg	NO	N																
Post	2013.09.13	53		103	A	P	Y	Y	N	F	DEPTH	depth	NO	N																
Post	2013.09.23	33	19.59	94	F	A	N	N	N	P				Y	N						2013-10-02	Y	0	N	N	94	N	N		18
Post	2013.10.07	45	22.06	93	F	A	Y	N	N	P				Y	P	BRB	Y	Y		100	2013-11-27	Y	1	N	N	91	N	N	200	20
Post	2013.10.07	44		114	F	P	N	N	N	P				N																
Post	2013.10.19	38	29.76	86	A	P	Y	Y	N	P				Y	N						2013-11-01	N	2	Y	N	85	N	N	55	25

Post	2013.10.26	40	24.22	140	F	A	N	N	N	P				Y	N					2013-11-13	Y	0	N	N	140	N	N	244	20
Post	2013.10.29	40		149	F	A	N	N	N	F	TOO MANY, SIZ	load	NO	N															
Post	2013.10.30	45	26.49	110	F	A	Y	P	Y	P				Y	P	BRB	Y	Y	100	2013-12-17	Y	3	N	Y	112	Y	N	188	41
Post	2013.11.05	41		89	A	P	Y	Y	Y	P				N															
Post	2013.11.06	54		129	F	P	N	N	N	F	DEPTH, SIZE	load, depth	NO	N															
Post	2013.11.06	42	20.43	98	A	P	Y	Y	Y	P				Y	P	RECTUM	Y		100	2013-12-18	Y	0	N	Y	98	N	Y	203	18
Post	2013.11.08	35	20.45	110	F	A	N	N	N	P				Y	N					2013-11-15	Y	0	N	Y	110	N	N	190	25
Post	2013.11.11	44	22.2	144	F	A	N	N	N	F	T2PERF+SIZE	load, t2perf	NO	N															
Post	2013.11.19	47	18.25	92	F	A	Y	Y	N	P				Y	Y	BRB	Y	Y	200	2013-11-29	Y	2	N	N	84	N	N	215	45
Post	2013.12.03	44	23.87	149	F	A	N	N	N	P				Y	N					2013-12-31	Y	2	N	N	149	N	N	295	16
Post	2013.12.06	45	24.77	101	F	P	Y	Y	Y	P				Y	Y	BRB	Y	Y	200	2014-01-14	Y	2	N	Y	101	N	N	195	39
Post	2013.12.10	39	19.92	108	FA	A	Y	Y	N	P				Y	Y	BRB	Y	Y	100	2013-12-24	Y	0	N	Y	108	N	N	241	29
Post	2013.12.11	33		91	A	P	Y	Y	Y	F	DEPTH	depth	NO	N															
Post	2013.12.13	46	19.81	143	F	A	N	N	N	P				Y	N					2014-01-28	Y	2	N	N	143	Y	N	297	18
Post	2013.12.13	36		91	F	A	N	N	Y	F	T2PERF+SIZE	load, t2perf	NO	N															
Post	2013.12.13	45		114	F	A	N	N	Y	P				N															
Post	2013.12.21	30	19.78	85	F	A	Y	Y	N	P				Y	Y	BRB	Y	Y	150	2014-01-07	Y	0	N	N	89	N	N	245	32
Post	2013.12.28	46	25.64	95	F	A	Y	N	N	P				Y	P	BRB	Y	Y	300	2014-01-21	Y	2	N	N	111	N	N	200	30
Post	2013.12.30	44	25.07	95	A	A	N	N	Y	P				Y	N					2014-01-15	N	2	N	Y	95	Y	N	175	25
Post	2014.01.03	43	21.1	101	FA	P	Y	Y	Y	P				Y	Y	BRB	Y	Y	150	2014-02-04	Y	2	N	Y	97	N	N	280	45
Post	2014.01.06	44	25.42	126	F	A	Y	N	N	P				Y	Y	BRB	Y	Y	200	2014-04-15	Y	1	Y	N	90	N	N	230	35
Post	2014.01.08	41	28.43	100	A	A	N	N	N	P				Y	N					2014-02-05	Y	1	N	N	100	N	N	212	17
Post	2014.01.09	41	18.36	119	F	A	N	N	N	P				Y	N					2014-01-29	Y	0	N	N	119	N	N	218	23
Post	2014.01.10	48	26.44	117	F	A	N	N	N	P				N															
Post	2014.01.14	39	21.74	239	F	A	N	N	N	P				Y	N					2014-02-26	Y	0	N	N	239	N	N	303	20
Post	2014.01.16	48	22.03	132	F	A	N	N	N	P				Y	N					2014-02-19	Y	3	N	N	132	Y	N	273	23
Post	2014.01.17	45		111	F	A	N	N	N	F	DEPTH	depth	NO	N															
Post	2014.01.20	47	25.03	120	A	A	N	N	Y	P				Y	N					2014-02-11	Y	2	N	Y	120	N	N	264	24
Post	2014.01.20	48	20.66	89	A	A	Y	Y	N	P				Y	N					2014-03-19	Y	0	N	N	89	N	N	223	23
Post	2014.01.22	42	28.64	131	F	A	N	N	N	P				Y	N					2014-02-18	Y	1	N	N	131	N	N	270	19
Post	2014.01.27	49	21.99	116	F	A	Y	Y	N	P				Y	N					2014-03-05	Y	2	N	N	116	N	N	165	20
Post	2014.02.07	46	23.04	116	F	A	N	N	N	P				Y	N					2014-03-12	Y	2	N	N	116	N	N	167	20
Post	2014.02.12	44	20.04	105	F	P	Y	Y	N	F	DEPTH	depth	NO	N															
Post	2014.02.13	46	29.55	195	FA	A	N	N	N	P				Y	N					2014-05-28	Y	0	Y	N	174	N	N	270	20
Post	2014.02.17	45	22.67	117	A	A	N	N	N	P				Y	N					2014-03-26	Y	2	N	N	117	N	N	255	22
Post	2014.02.20	39	19.37	147	F	A	N	N	N	P				Y	N					2014-05-20	Y	0	Y	Y	122	N	N	165	43
Post	2014.02.21	46	20.5	102	A	A	N	N	Y	P				Y	N					2014-04-09	Y	2	N	Y	102	N	N	180	31
Post	2014.02.24	43		211	F	A	N	N	N	F	TOO LARGE	load	NO	N															
Post	2014.02.25	45	24.65	126	F	A	N	N	N	P				Y	N					2014-04-29	N	3	N	N	126	Y	N	117	20
Post	2014.03.14	44	23.9	118	F	A	N	N	N	P				Y	N					2014-04-23	Y	2	N	N	118	N	N	290	20
Post	2014.03.14	43	19.78	100	F	P	Y	Y	Y	P				Y	Y	BRB	Y	Y	250	2014-06-17	Y	1	N	Y	103	N	N	240	34
Post	2014.03.18	43	19.7	95	F	A	N	N	Y	P				Y	N					2014-04-16	Y	0	N	Y	95	Y	N	235	25
Post	2014.03.28	49	25.01	134	F	A	N	N	N	P				Y	N					2014-04-30	Y	2	N	N	134	N	N	215	20

Post	2014.03.28	42	22.04	108	F	P	Y	Y	N	P				Y	Y	BRB	N	N		400	2014-06-03	N	2	Y	N	76			119	
Post	2014.03.29	43	25.74	107	A	A	N	N	Y	P				Y	N						2014-07-29	Y	2	N	Y	107	N	N	200	20
Post	2014.03.31	43	25.09	98	F	A	Y	Y	N	P				Y	Y	BRB	Y	Y		150	2014-07-15	Y	1	N	N	103	N	N	235	27
Post	2014.04.01	46		153	F	A	N	N	Y	P				N																
Post	2014.04.02	41	21.57	137	F	A	N	N	Y	P				Y	N						2014-06-25	Y	2	N	Y	137	N	N	245	26
Post	2014.04.07	44	21.64	136	FA	A	N	N	N	P				Y	N						2014-05-14	Y	2	N	N	136	N	N	240	22
Post	2014.04.11	41	18.12	121	F	A	Y	P	N	P				Y	P	BRB	Y	Y		100	2014-07-16	Y	0	N	N	140	N	N	265	28
Post	2014.04.18	46	22.71	135	F	A	N	N	N	P				Y	N						2014-05-21	Y	0	N	N	135	Y	N	248	19
Post	2014.04.18	42	21.34	99	F	A	Y	Y	N	P				Y	Y	BRB	Y	Y		100	2014-07-09	Y	2	N	N	96	N	N	135	24
Post	2014.04.21	43	21.56	97	F	P	Y	Y	N	P				Y	Y	THRU BL	Y	N	500	250	2014-09-03	Y	2	Y	N	86	Y	N	200	44
Post	2014.04.21	46		153	F	A	N	N	N	F	TOO MANY, SIZ	load	NO	N																
Post	2014.04.22	39	25.52	107	F	A	Y	P	Y	P				Y	P	BRB	Y	Y		150	2014-05-07	Y	0	Y	Y	112	N	N	174	22
Post	2014.04.25	34	22.75	106	A	A	N	N	Y	F	PERF (OTHER PA	other pathol	NO	N																
Post	2014.04.26	44	21.22	136	F	A	N	N	N	P				Y	N						2014-07-30	Y	0	N	N	136	N	N	230	19
Post	2014.04.29	43	20.55	127	F	A	N	N	Y	P				Y	N						2014-06-18	Y	1	N	Y	127	N	N	250	25
Post	2014.04.29	42	18.83	136	F	A	N	N	Y	P				Y	N						2014-07-01	Y	0	N	N	136	Y	N	280	22
Post	2014.05.13	41	23.73	85	F	A	Y	Y	N	P				Y	Y	THRU BL	Y	N		200	2014-07-02	Y	0	N	N	83	N	N	150	45
Post	2014.05.14	39	19.94	84	A	A	Y	Y	N	P				Y	Y	THRU BL	Y	N	500	150	2014-07-23	Y	0	Y	N	75	Y	N	193	29
Post	2014.05.16	46		128	F	A	N	N	N	F	DEPTH	depth	NO	N																
Post	2014.05.22	34	19.83	108	F	P	Y	Y	N	P				Y	Y	BRB	Y	Y		200	2014-08-05	Y	0	N	N	110	N	N	223	25
Post	2014.05.26	39		94	FA	A	Y	Y	N	P				N																
Post	2014.05.28	44	20.86	120	F	A	Y	Y	N	P				Y	Y	BRB+ma	Y	Y	500	100	2014-08-13	Y	0	N	N	122	N	N	258	40
Post	2014.06.13	47	26.22	171	FA	A	N	N	N	P				Y	N						2014-10-01	Y	1	Y	N	113	N	N	215	25
Post	2014.06.13	47		186	F	A	N	N	N	F	FAT, SIZE, NUM	load, fat	NO	N																
Post	2014.06.16	40		111	F	A	Y	Y	N	P				N																
Post	2014.06.18	44	19.07	110	FA	A	N	N	Y	P				Y	N						2014-08-20	Y	2	N	Y	110	N	N	233	27
Post	2014.06.20	47	17.71	95	F	P	Y	Y	Y	F	DEPTH	depth	NO	N																
Post	2014.06.23	33	16.16	92	A	P	Y	Y	N	P				Y	Y	BRB	Y	Y	400	300	2014-08-19	Y	0	N	N	88	N	N	268	34
Post	2014.06.24	45	20.44	110	FA	A	Y	N	N	P				Y	Y	BRB	Y	Y	300	200	2014-09-24	Y	1	Y	N	94	N	N	270	25
Post	2014.06.27	47	24.52	127	A	A	Y	Y	N	P				Y	N						2014-07-08	Y	2	N	N	127	N	N	205	23
Post	2014.07.03	34		121	F	A	N	N	N	P				N																
Post	2014.07.04	36		129	A	A	N	N	N	F	TOO LARGE	load	NO	N																
Post	2014.07.05	54		145	A	A	N	N	Y	F	TOO MANY, SIZ	load	NO	N																
Post	2014.07.07	43	30.84	150	F	A	N	N	Y	P				Y	N						2014-10-07	Y	2	Y	Y	113	Y	Y	255	26
Post	2014.07.07	49	28.52	104	A	A	N	N	Y	F	FAT, PERF	fat, t2perf	NO	N																
Post	2014.07.10	39	20.01	138	F	A	N	N	N	F	DEG	deg	NO	N																
Post	2014.07.11	36	20.04	80	A	A	Y	P	Y	P				Y	P	BRB	Y	Y		150	2014-12-10	Y	1	Y	Y	77	N	N	260	43
Post	2014.07.12	43		129	F	A	N	N	N	F	TOO MANY, SIZ	load	NO	N																
Post	2014.07.14	44	20.31	137	F	A	N	N	N	P				N																
Post	2014.07.17	46	18.79	104	F	A	Y	Y	N	P				Y	Y	BRB	Y	Y	500	100	2014-10-08	Y	3	Y	N	96	N	N	202	27
Post	2014.07.18	48	22.36	100	F	P	Y	Y	N	F	DEPTH, OTHER	other pathol	NO	N																
Post	2014.07.18	45		100	F	P	Y	Y	N	F	T2PERF	t2perf	NO	N																
Post	2014.07.23	42	18.03	109	A	A	Y	N	N	P				Y	Y	BRB	Y	Y	500	150	2014-09-30	Y	0	Y	N	74	N	N	180	36

Post	2014.07.28	47		98	A	P	Y	Y	N	F	DEPTH	depth	NO	N																
Post	2014.08.06	55		153	F	A	N	N	N	F	SIZE, DEPTH	load, depth	NO	N																
Post	2014.08.07	43	20.43	95	F	A	Y	P	Y	P				Y	N						2014-11-04	Y	2	N	Y	95	N	N	170	30
Post	2014.08.08	38	21.05	98	A	A	N	N	Y	P				Y	N						2014-10-21	Y	2	N	Y	98	N	N	227	31
Post	2014.08.12	45	22.46	85	A	A	Y	Y	N	F	DEPTH	depth	NO	N																
Post	2014.08.13	42	23.05	129	F	A	Y	N	N	P				Y	Y	BRB	Y	Y	400	150	2014-11-18	Y	2	Y	N	103	N	N	305	45
Post	2014.08.20	50	19.47	95	F	A	N	N	Y	P				Y	N						2014-11-12	Y	2	N	Y	95	N	N	68	20
Post	2014.08.21	46	18.73	125	F	A	Y	Y	N	P				Y	Y	BRB	Y	Y		100	2014-09-16	Y	2	N	N	115	N	N	247	33
Post	2014.08.22	45	26.22	102	F	A	N	N	Y	P				Y	N						2014-10-29	Y	2	N	Y	102	N	N	92	22
Post	2014.08.25	53	22.39	158	F	A	N	N	N	P				Y	N						2014-11-19	Y	2	Y	N	122	N	N	223	14
Post	2014.08.26	38	21.97	108	F	A	Y	Y	Y	P				Y	Y	BRB	Y	Y	500	100	2014-10-22	Y	0	N	Y	93	N	N	160	23
Post	2014.09.01	49	22.43	116	F	A	Y	Y	N	P				Y	Y	BRB	Y	Y	500	100	2014-11-26	Y	3	N	N	119	N	N	158	25
Post	2014.09.11	39		133	F	A	N	N	N	F	DEPTH, TOO MA	load, depth	NO	N																
Post	2014.09.11	50	23.46	146	F	A	N	N	N	F	T2PERF+SIZE	load, t2perf	NO	N																
Post	2014.09.12	44	28.05	86	F	A	Y	Y	N	P				Y	Y	BRB	Y	Y	500	200	2014-12-16	Y	0	Y	N	74	N	N	220	35
Post	2014.09.14	38		123	F	A	N	N	Y	F	TOO MANY, SUI	surgical mate	NO	N																
Post	2014.09.16	48	24.74	117	F	A	N	N	N	P				N																
Post	2014.09.17	38	21.08	109	F	A	N	N	N	P				Y	N						2015-01-06	Y	0	N	N	109	N	N	215	25
Post	2014.09.22	47	20.53	100	F	A	Y	Y	N	P				Y	Y	BRB+ma:	Y	Y	500	350	2014-12-17	Y	2	N	N	98	N	N	135	52
Post	2014.09.26	47	23.81	125	F	A	N	N	N	P				Y	N						2015-01-07	N	2	Y	N	109	N	N	80	16
Post	2014.09.30	29	25.4	92	F	P	Y	Y	N	P				Y	Y	THRU BL	Y	N	500	400	2015-01-20	N	0	Y	N	77	N	N	150	30
Post	2014.10.05	42	23.66	108	F	A	N	N	N	P				N																
Post	2014.10.07	45	24.6	171	F	A	N	N	N	P				Y	N						2014-12-24	Y	0	N	N	171	N	N	260	28
Post	2014.10.08	45	19.24	113	F	A	N	N	N	P				Y	N						2015-01-21	Y	0	Y	N	137	Y	N	265	20
Post	2014.10.10	46	20.02	138	F	A	Y	N	Y	P				Y	Y	BRB	N	N	500	400	2015-01-28	N	2	Y	Y	89			110	
Post	2014.10.15	48	25.96	108	F	A	Y	P	N	P				Y	Y	BRB	Y	Y	500	200	2015-02-03	Y	1	N	Y	113	N	N	200	37
Post	2014.10.16	46		104	F	P	Y	Y	Y	F	DEPTH	depth	NO	N																
Post	2014.10.16	48	19.72	123	F	A	N	N	N	F	T2PERF	t2perf	NO	N																
Post	2014.10.17	35	23.27	108	F	A	Y	P	N	P				Y	N						2015-02-04	Y	0	N	N	108	N	N	225	20
Post	2014.10.28	47	21.94	99	F	A	Y	P	Y	P				Y	Y	THRU BL	Y	N	500	200	2015-02-24	Y	1	Y	Y	81	Y	N	240	43
Post	2014.10.30	51		142	F	A	N	N	N	F	SIZE, TOO MAN`	load	NO	N																
Post	2014.11.05	49	22.1	164	F	A	N	N	N	P				Y	N						2015-03-04	Y	2	Y	N	130	N	N	275	27
Post	2014.11.07	42	19.56	101	F	A	N	N	N	P				Y	N						2015-03-03	Y	0	N	N	101	N	N	245	28
Post	2014.12.29	44	22.38	104	F	P	Y	Y	N	P				Y	Y	THRU BL	Y	N	400	300	2015-01-13	Y	1	N	N	105	N	N	210	53
		43	22.54	119																173.8						119.2			218	30
		5	2.739	25.7																85.74						27			49.2	10.6
		25	16.16	72																100						68			55	13
		55	32.01	239																400						239			329	83
